# Conserved Prosegment Residues Stabilize a Late-Stage Folding Transition State of Pepsin Independently of Ground States

**DOI:** 10.1371/journal.pone.0101339

**Published:** 2014-07-01

**Authors:** Derek R. Dee, Yasumi Horimoto, Rickey Y. Yada

**Affiliations:** 1 Biophysics Interdepartmental Group, University of Guelph, Guelph, Ontario, Canada; 2 Department of Food Science, University of Guelph, Guelph, Ontario, Canada; Universidad de Granada, Spain

## Abstract

The native folding of certain zymogen-derived enzymes is completely dependent upon a prosegment domain to stabilize the folding transition state, thereby catalyzing the folding reaction. Generally little is known about how the prosegment accomplishes this task. It was previously shown that the prosegment catalyzes a late-stage folding transition between a stable misfolded state and the native state of pepsin. In this study, the contributions of specific prosegment residues to catalyzing pepsin folding were investigated by introducing individual Ala substitutions and measuring the effects on the bimolecular folding reaction between the prosegment peptide and pepsin. The effects of mutations on the free energies of the individual misfolded and native ground states and the transition state were compared using measurements of prosegment-pepsin binding and folding kinetics. Five out of the seven prosegment residues examined yielded relatively large kinetic effects and minimal ground state perturbations upon mutation, findings which indicate that these residues form strengthened and/or non-native contacts in the transition state. These five residues are semi- to strictly conserved, while only a non-conserved residue had no kinetic effect. One conserved residue was shown to form native structure in the transition state. These results indicated that the prosegment, which is only 44 residues long, has evolved a high density of contacts that preferentially stabilize the folding transition state over the ground states. It is postulated that the prosegment forms extensive non-native contacts during the process of catalyzing correct inter- and intra-domain contacts during the final stages of folding. These results have implications for understanding the folding of multi-domain proteins and for the evolution of prosegment-catalyzed folding.

## Introduction

**Figure 1:**
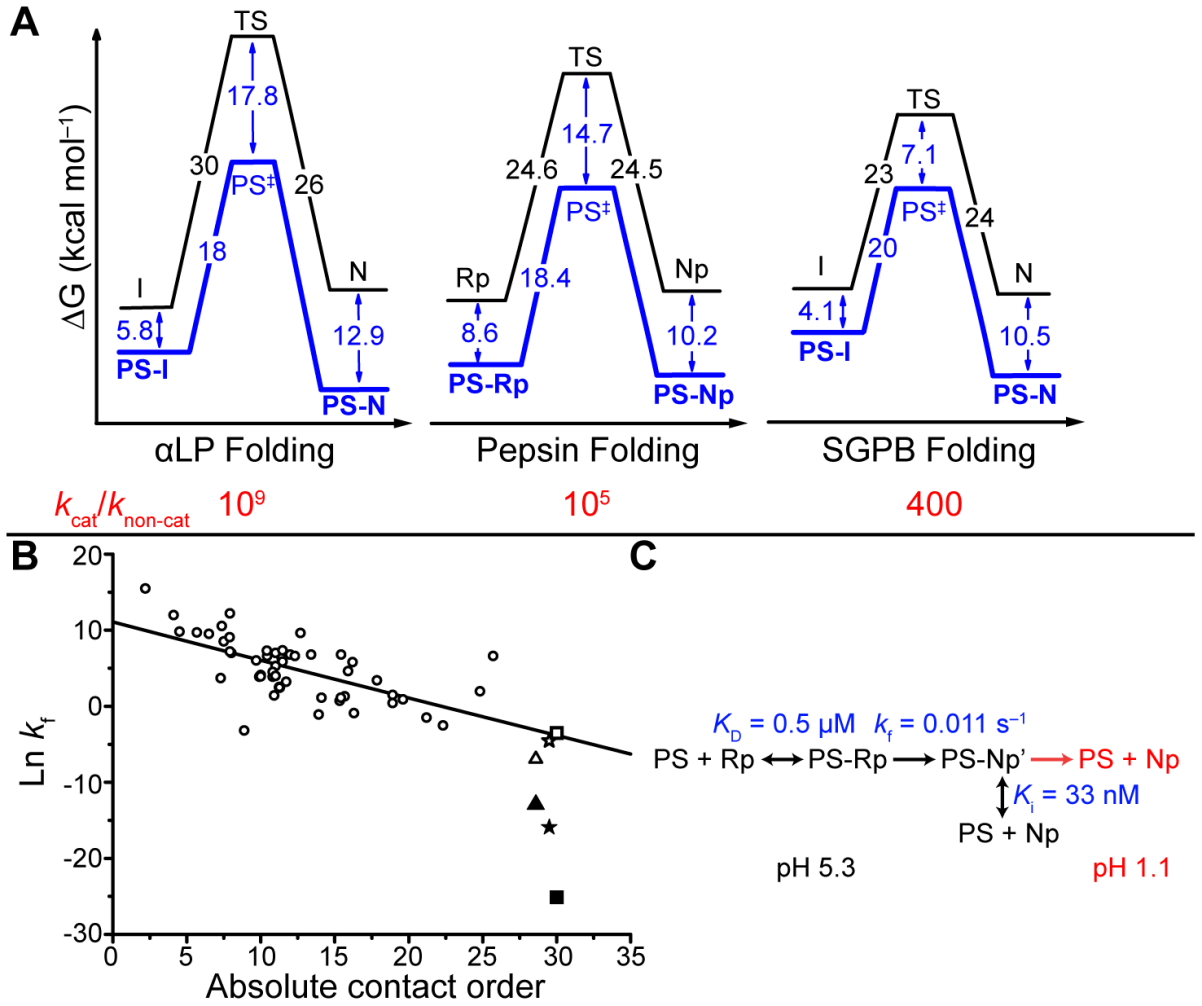
Zymogen-derived proteins deviate from common trends in protein folding. (A) Comparison of the non- and PS-catalyzed folding of αLP [8], SGPB [5] and pepsin [7]. (B) Relation between topology and folding rate for a number of two- and three-state folding proteins (circles, data taken from [3], [21], [22]). The folding rate of αLP (squares), SGPB (triangles) and pepsin (stars) is accelerated to the value (hollow points) expected based on the topology, only when the PS is included. (C) Reaction scheme of pepsin PS-catalyzed folding. The PS binds Rp and catalyzes its conversion to Np at pH 5.3, where the PS is a strong inhibitor of Np. The PS dissociates from Np at pH<3.

General thermodynamic and kinetic features of protein folding are known, such as that the natively folded conformation of a protein is thermodynamically stabilized [1] and that there is a relationship between topology and folding rate, which holds for both two-state and multi-state folding proteins [2], [3]. However, zymogen-derived proteins which require a prosegment (PS) domain to catalyze folding, such as the serine peptidases αLP [4], SGPB [5], and subtilisin [6], and the aspartic peptidase pepsin [7], deviate from the common thermodynamic and kinetic trends in protein folding to varying degrees. For example, as shown in **Fig 1A**, the native states of αLP, pepsin and SGPB are thermodynamically unstable (Δ*G*_N-I_  = −4 kcal/mol) [8], metastable (Δ*G*_N-I_ = −0.1 kcal/mol) [7] and marginally stable (Δ*G*_N-I_ = +1 kcal/mol) [9], relative to an intermediate state, respectively. Additionally, in the absence of the PS domain, these proteins fold much slower than would be estimated based on their topology (**Fig 1B**).

When the PS is included these deviations are corrected: the PS shifts the folding equilibrium towards the PS-native state complex and catalyzes folding by stabilizing the folding transition state (TS) (bold, blue line in **Fig 1A**). Once folding is complete, the PS is removed and the folding and unfolding activation barriers increase, leaving behind a kinetically trapped native state (black line in **Fig 1A**). There is an intriguing separation of low- and high-barrier folding landscapes, with and without the PS, respectively, and understanding how a PS domain stabilizes the folding TS should be informative for understanding kinetic folding/unfolding barriers in general, which remain poorly understood [10]. Despite several studies of PS-catalyzed folding [11]–[14], the mechanism by which a PS stabilizes the TS remains unknown.

Pepsin, which is derived from its zymogen pepsinogen, folds to a thermodynamically stable yet non-native form (termed refolded pepsin, Rp) upon removal from denaturing conditions [15]. Rp is inactive, contains native-like secondary and tertiary structure and has a greater thermal stability (Δ*T*_m_ = +5°C) [15] and reduced picosecond diffusive motions when compared to native pepsin (Np) [16]. These features suggest that Rp is a late-stage folding intermediate, which in turn indicates that the PS operates late in the pepsinogen folding pathway.

The folding of Rp to Np serves as a useful model for examining late-stage folding transitions between compact-misfolded and native states, and under native conditions (*i.e*., pH 5.3 and no denaturants), which are often difficult to access experimentally. Indeed, the study of such misfolding events is often only possible at the single-molecule level [17]. Given the increased risk of misfolding in multi-domain proteins, via incorrect domain-domain contacts [18], [19], it is prudent to examine the various mechanisms by which proteins have evolved to avoid such issues.

The present study undertook to examine the energy landscape of PS-catalyzed pepsin folding by measuring the contributions of specific PS residues to stabilizing the folding TS and ground states (Rp and Np). Our method took advantage of the bimolecular PS-catalyzed folding reaction of pepsin (**Fig 1C**). Synthetic PS peptide was added exogenously to Rp and Np and the changes in equilibrium stability of PS-Np relative to PS-Rp upon mutation, ΔΔ*G_PS(Np-Rp)_*, were determined from the difference in the changes in binding energies, as shown in **Fig S1**. Changes in activation energy upon mutation, ΔΔ*G^‡^*, were determined by measuring changes in the rate of PS-catalyzed folding.

A simple comparison of ΔΔ*G^‡^* and ΔΔ*G_PS(Np-Rp)_* yields the so-called Φ-value [20]. Φ-values are calculated as the ratio of the change in activation energy to the change in equilibrium stability (Φ =  ΔΔ*G^‡^*/ΔΔ*G*_N-D_) upon introducing a point mutation. Under the classical interpretation, a residue can belong to a region of either unfolded or native structure in the TS, giving rise to the limiting Φ-values of 0 or 1 [23]; however, a range of fractional Φ-values are more commonly observed [24], [25]. Φ-value analysis is often applied to studies of unimolecular folding [26]–[31], in which all energies are measured relative to one state, generally the unfolded state (and thus mutation effects on this state go unresolved [32]). Compared to Φ-value analysis of unimolecular folding, the bimolecular approach allows a comparison of the effects of point mutants on each individual ground state and the TS. The ΔΔ*G* values obtained for each individual state are more informative than the Φ-values derived from them, yet Φ-values are also reported here as a point of comparison.

Application of Φ-value analysis to the PS-catalyzed folding of pepsin yielded predominantly abnormal Φ-values (Φ>1, Φ<0), reflecting the finding that most of the mutations resulted in a greater destabilization of the PS-TS than of either PS-Rp or PS-Np. This greater sensitivity to perturbation of the PS-TS complex likely indicates either the presence of strong non-native interactions or reduced conformational strain in the TS, or a combination of both factors.

## Results

### Selection of point mutations

**Figure 2:**
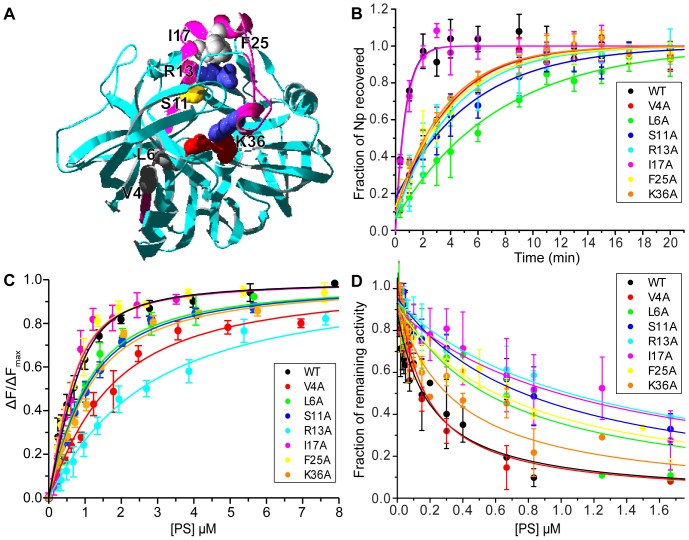
Effects of PS point mutants on binding and catalyzing pepsin folding. (A) Structure of pepsinogen (PDB code: 3PSG) with the PS (pink) located between the N- and C-terminal lobes, forming part of a six-stranded β-sheet, and K36 of the PS interacts with the catalytic residues, D32 and D215 (red). PS residues selected for mutation to Ala are shown in space-filling form and coloured according to type (grey-hydrophobic, orange-polar, blue-basic, red-acidic). (B) Comparison of wild-type and mutant PS-catalyzed folding of pepsin. The rate of PS-catalyzed folding (*k*_f_) was determined by adding PS to Rp, at pH 5.3, 15°C (see **Text S2**: folding rate followed Arrhenius temp-dependence from 0—15°C, shown in **Fig S5**), and measuring the formation of Np based on enzyme activity measured at pH 1.2, 25°C. The data were fit according to a monoexponential function to obtain *k*_f_. (C) Comparison of wild-type and mutant PS affinity for Rp. PS-Rp binding was determined by following the increase in Trp-fluorescence of pepsin as a function of [PS]. The data were fit according to eq 1 to determine the dissociation constant, *K*_d_, at 20°C, pH 5.3. (d) Comparison of wild-type and mutant PS affinity for Np. The reduction in Np activity was measured as a function of [PS]. The data were fit according to a competitive inhibitor model, eq 2, to determine the inhibition (dissociation) constant, *K*_i_, at 20°C, pH 5.3. All data are reported as the average ± SD of 3-5 measurements for each PS peptide.

PS mutations were chosen on the basis of examining sequence conservation and the available zymogen crystal structures of porcine pepsinogen and human progastricsin (**Fig S2**). As proteins are believed to have evolved stable, mutually supportive native contacts to avoid degeneracy on the folding landscape (*i.e*., minimally frustrated contacts) [33], highly conserved residues may be particularly important for proper folding. A conserved domain search within the Pfam database [34] revealed that PS residues 1–29 of pepsinogen correspond to the A1 propeptide conserved motif, which is found in 402 sequences from 107 animal species. Pepsinogen homologues were identified using a sequence similarity search and these were further aligned, focusing on the 44-residue PS domain, revealing that PS residues L6 and R13 are strictly conserved (**Fig S3**). Only the R13 and K36 side-chains of the PS form hydrogen bonds with the mature pepsin domain, with hydrogen bonds formed between R13 and pepsin D11 and between K36 and the catalytic residues of pepsin, D32 and D215. Seven residues were chosen for mutation, shown in **Fig 2A**: the strictly conserved L6 and R13, the semi-conserved V4, S11, F25 and K36, and the non-conserved I17. F25 was chosen primarily to probe the effect of mutating the second α-helical segment, which makes no direct contacts with the pepsin domain, such that the N-terminal β-strand and all three α-helical segments of the PS were probed with mutations.

The residues were replaced with Ala in the corresponding seven synthetic peptides. Ala substitutions were shown to be generally conservative mutations that do not introduce non-native interactions, which would further complicate data analysis, while at the same time provide a measurable destabilization [32].

### Effects of PS mutants on binding and folding catalysis

The PS-catalyzed folding and binding data are summarized in **Fig's 2** and **S4** and **Table 1**. All of the mutants markedly slowed the rate of PS-catalyzed folding except for I17A, which had no effect. The L6A mutant gave the slowest folding rate, while the other mutants resulted in similar rates. The narrow range of effects that mutations had on the folding rate suggests that the PS stabilizes the folding TS via contacts made along an extensive portion of the PS and not from localized contacts. For some of the mutants, *e.g*. S11A, a burst-phase in the folding kinetics was noticeable (**Fig S4**), although the basis for this feature is not yet clear. Generally the PS mutants had a small impact on PS affinity for Rp. One exception was R13A, which reduced the binding affinity 7-fold, while the effects of the other mutants were modest (2 to 3-fold reduction in affinity) or had no effect at all (I17A and F25A were similar to PS_wt_). The mutations resulted in a wider distribution of affinities for Np, measured by PS inhibition of Np. In this case, V4A was similar to PS_wt_ while R13A and I17A resulted in the largest reduction in affinities for Np.

**Table 1:** Changes in binding and folding constants^a^ and associated free energies^b^ upon mutation of the PS.

PS-mutant	*k*_f_ (min^-1^)	*K*_d_ (µM)	*K*_i_ (nM)	ΔΔ*G_PS-Rp_*	ΔΔ*G^‡^*	ΔΔ*G_PS-Np_*	ΔΔ*G_PS(Np-Rp)_*	Φ
WT	1.42±0.34	0.31±0.03	64.8±11.4					
V4A	0.27±0.03	1.19±0.23	50.9±6.9	0.79±0.12	0.94±0.15	−0.14±0.13	−0.93±0.18	−1.0±0.3
L6A	0.13±0.01	0.64±0.09	193.5±38.8	0.43±0.10	1.36±0.15	0.64±0.16	0.20±0.18	6.6±6.0
S11A	0.18±0.02	0.68±0.07	270.1±31.5	0.47±0.08	1.18±0.15	0.83±0.12	0.36±0.15	3.2±1.4
R13A	0.24±0.02	1.96±0.29	382.2±37.2	1.08±0.10	1.02±0.15	1.03±0.12	−0.05±0.15	−21±67
I17A	1.40±0.26	0.21±0.09	393.8±65.1	−0.21±0.24	0.01±0.18	1.05±0.14	1.26±0.28	0.0±0.1
F25A	0.26±0.04	0.25±0.03	230.4±29.1	−0.12±0.08	0.96±0.17	0.74±0.13	0.86±0.15	1.1±0.3
K36A	0.28±0.03	0.72±0.14	113.3±20.3	0.50±0.13	0.94±0.15	0.33±0.15	−0.17±0.19	−5.5±6.4

a Folding rate constants were measured at 15°C while binding constants were determined at 20°C. Data are given as the mean ± SD obtained from non-linear curve fitting.

b Free energy units are in kcal/mol with ± SD derived by propagation of errors.

### Changes in the PS-catalyzed folding energy landscape

**Figure 3:**
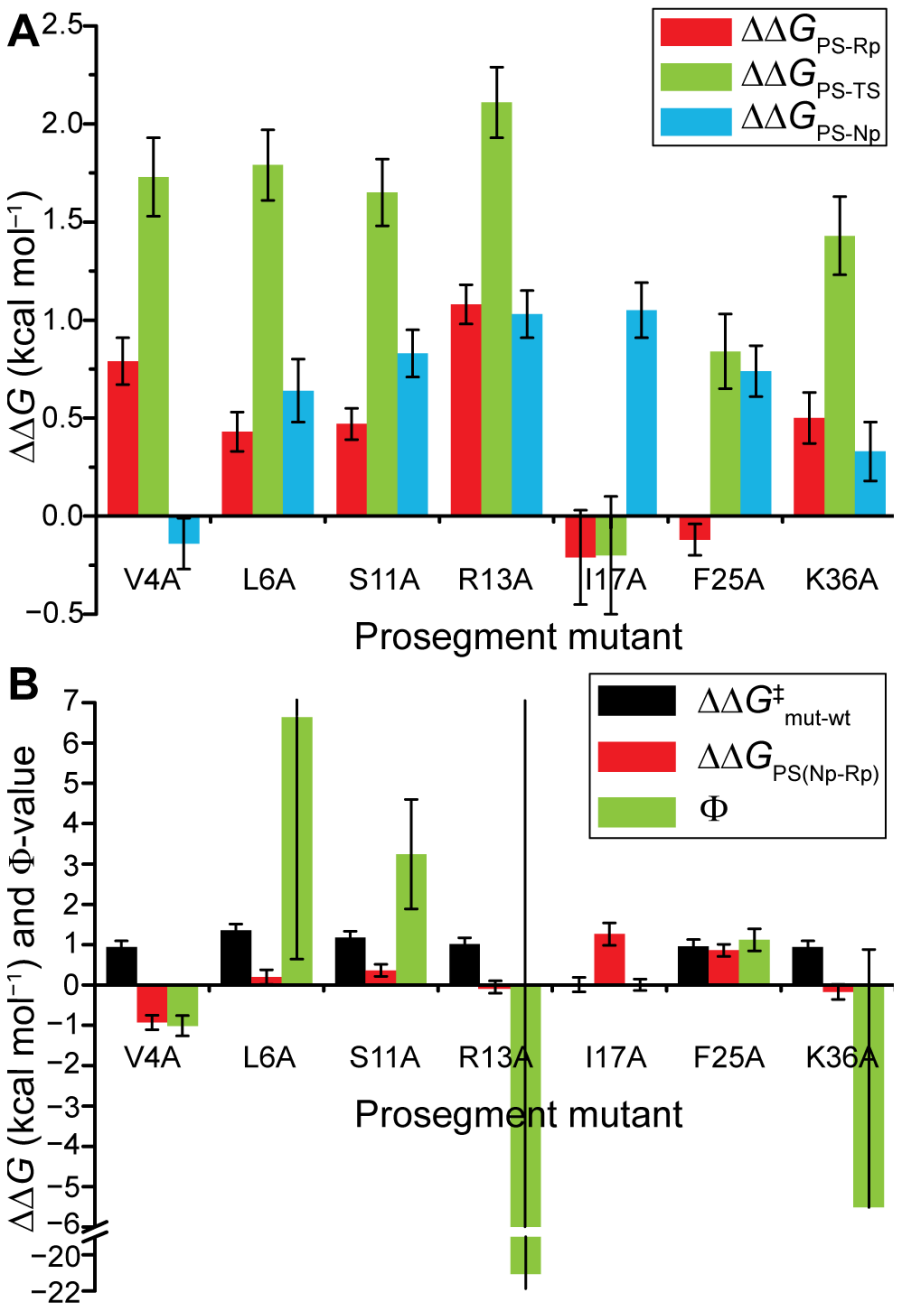
Changes in the PS-catalyzed folding energy landscape upon mutation of the PS peptide. (A) The changes in energy of each conformation were determined as changes in binding energies. (B) Φ-values calculated from the ratio of the changes in activation energy (ΔΔ*G^‡^*) and free energy difference between PS-Np and PS-Rp (ΔΔ*G_PS(Np-Rp)_*). Error bars show ± SD derived by propagation of errors.

The changes in PS-Rp and PS-Np binding energy upon mutation (ΔΔ*G_PS-Rp_* and ΔΔ*G_PS-Np_*) were obtained from measurements of *K*_d_ and *K*_i_. The change in PS-TS binding energy (ΔΔ*G_PS-TS_*) was taken as the change in the folding activation energy, ΔΔ*G^‡^*, added to ΔΔ*G_PS-Rp_*, as Δ*G^‡^* was determined relative to PS-Rp. The changes in binding and folding activation energies are given in **Table 1**. The effects of each PS mutation on the PS-catalyzed folding landscape are readily compared by plotting the changes in binding energies of the denatured (PS-Rp), transition (PS-TS) and native (PS-Np) states, as shown in **Fig 3A**.

PS-Rp was destabilized by mutations at V4, L6, S11 and R13, which corresponds to the N-terminal β-strand and α-helix-1 of the PS within pepsinogen (**Fig 2A**), indicating that this region may play a dominant role in defining the initial PS-Rp complex. Conversely, both I17A and F25A had a negligible effect on PS-Rp binding, indicating that the PS is likely unstructured in this region within PS-Rp. All of the mutations, except V4A, R13A, and K36A, were more destabilizing to PS-Np than to PS-Rp. V4A had a very small stabilizing effect on PS-Np and a relatively large destabilizing effect on PS-Rp. Conversely, R13A destabilized both the PS-Np and PS-Rp complexes – the similar magnitudes of ΔΔ*G_PS-Rp_* and ΔΔ*G_PS-Np_* suggest that R13 had similar contacts in both PS-Rp and PS-Np. K36A was also nearly equally destabilizing to PS-Rp and PS-Np, suggesting a similar structure in both complexes, although overall K36A had less of an impact on binding to Rp and Np than R13A.

With the exception of I17A, the mutations were most destabilizing to the PS-TS complex, particularly the mutation of the strictly conserved R13. I17A had a negligible effect on PS-Rp and PS-TS stability, yet was one of the most destabilizing mutations to the PS-Np complex, indicating that this non-conserved residue makes no contribution to catalyzing folding but contributes to driving the equilibrium towards PS-Np.

Φ-values were obtained, using the data in **Fig 3A**, by subtracting ΔΔ*G_PS-Rp_* from both ΔΔ*G_PS-TS_* (to obtain ΔΔ*G^‡^*) and ΔΔ*G_PS-Np_* (to obtain ΔΔ*G_PS(Np-Rp)_*), and then dividing the first result by the second (**Fig 3B**). A Φ-value close to either 0 or 1 would indicate that a PS residue adopts a conformation identical to that in either PS-Rp or PS-Np in the PS-TS complex, respectively. As seen in **Table 1** and **Fig 3B**, most of the mutants gave rise to large positive or negative Φ-values due to the relatively small ΔΔ*G_PS(Np-Rp)_* and large ΔΔ*G^‡^* values. L6A, R13A and K36A, in particular, yielded exceptionally large Φ-values (as are the associated errors) owing to ΔΔ*G_PS(Np-Rp)_* close to zero while ΔΔ*G^‡^* is ∼1 kcal/mol. As discussed below, these values likely reflect the formation of non-native interactions and/or reduced conformational strain in the TS.

## Discussion

### PS stabilizes TS independently of ground states

**Figure 4:**
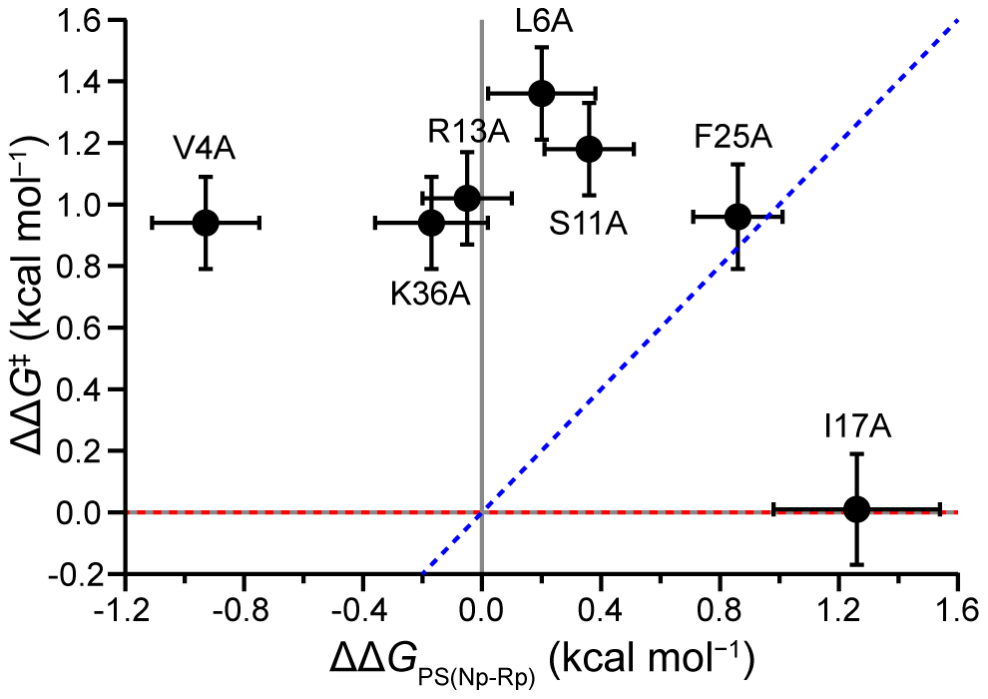
Brønsted plot. A comparison of the mutation effects on the folding activation energy as a function of the change in equilibrium stability. Dashed lines indicate the trend lines for ΔΔ*G* values that would give rise to Φ-values of 0 or 1 and error bars show ± SD.

For small, two-state folding proteins, a predominance of low fractional Φ-values is interpreted as a diffuse TS with weakened native-like structure, while Φ-values clustering towards low and high fractional values is interpreted as a polarized TS structure, with some regions forming native-like contacts and others being unfolded [35]. The Φ-values presented for PS-pepsin show a very different trend: instead of fractional values, five of the seven residues were characterized by large positive or negative Φ-values indicating a highly structured TS state with strengthened interactions. A useful means by which to compare the kinetic effect of various mutations is to use a Brønsted plot [35], as shown in **Fig 4**. It can be seen that the increase in folding activation energy occurs independently of the ground state perturbation, indicating that all of the PS residues examined, except for I17, play a common role in defining the folding barrier. The R13A and K36A mutations gave particularly large kinetic effects, with ΔΔ*G_PS(Np-Rp)_* close to 0 and ΔΔ*G^‡^* of ∼1 kcal/mol. As strong kinetic effects were seldom observed out of a comparison of hundreds of mutations from various small, single domain proteins [24], [35], this data supports the idea that the PS plays a unique role in stabilizing the folding TS.

### Physical basis for large kinetic effects and abnormal Φ-values

The perturbations introduced by each mutation are characterized in most detail by the individual binding energy changes, ΔΔ*G_PS-Rp_*, ΔΔ*G_PS-Np_* and ΔΔ*G_PS-TS_* (**Fig 3A**). Ala-scanning allowed for the identification of key residues that provide extra PS-TS stabilization, yet the nature of these interactions remains open to speculation. A consequence of the larger and opposite effects on the TS compared to the ground states (**Fig 4**) is that this gives rise to Φ-values that fall outside the typical range of 0 to 1. When interpreting the large kinetic effects observed in PS-catalyzed pepsin folding, it is worth considering previous reports of abnormal Φ-values determined for unimolecular folding.

Although Φ-values outside the range 0 to 1 account for as much as 10 to 20% of those reported [23], it was argued that many of these unusual Φ-values are not reliable as they are associated with small ΔΔ*G*_N-D_ and ΔΔ*G^‡^* values [35]. In practice, what is considered the lower limit of ΔΔ*G*_N-D_ from which reliable Φ-values may be calculated differs among reports, ranging from 1.7 [35] to 0.6 [36] to 0.2 kcal/mol [30]. Fortunately, analysis of pepsin PS-catalyzed folding did not rely on direct measurements of the generally small values of ΔΔ*G_PS(Np-Rp)_*, as the individual binding energies provide specific detail on the perturbations introduced upon mutation to each of the PS-Rp and PS-Np complexes (**Fig 3A**). Even in the cases where ΔΔ*G_PS(Np-Rp)_* is close to 0 (*e.g*., L6A, R13A and K36A), the ΔΔ*G^‡^* values are substantial (∼1 kcal/mol) such that the associated Φ-values can be reliably classified as ‘abnormal’ (outside the range 0 to 1), even if they cannot be measured quantitatively owing to the correspondingly large errors. For example, R13A yields a Φ-value of –21±67 that is unreliable statistically, yet the underlying comparison that the Φ-value represents, that ΔΔ*G^‡^*  = 1.02±0.15> ΔΔ*G_PS(Np-Rp)_*  = −0.05±0.15 (all units in kcal/mol), is reliable.

It was previously observed that abnormal Φ-values occur more frequently for mutations that could lead to changes in stability or configurational dynamics of the denatured state, such as mutation of charged and polar residues or of Ala and Gly [37]. As the pepsin PS is unstructured on its own, as verified by CD (**Fig S6**), mutations would have minimal effects on the structure and stability of the PS, and the measured changes in binding energies can be ascribed entirely to changes in PS-pepsin interactions.

An analysis of 806 mutants from 24 proteins indicated that Φ-values are strongly influenced by packing density and local interactions [24]. Residues at the surface tend to make fewer and more localized contacts than internal residues and thus can adopt a native-like structure when only a few local contacts are formed. Furthermore, the ΔΔ*G*_N-D_ values for mutations at locations with few contacts are generally smaller than those for buried residues with many native contacts. Thus, mutations of surface residues result in both smaller ΔΔ*G*_N-D_ and larger Φ-values, while mutations at core residues tend to give larger ΔΔ*G*_N-D_ and smaller Φ-values [24], [37]. The PS residues examined in the present study are mostly buried from the solvent yet are not core residues, as the PS sits at the surface (**Fig 3A**). Fewer native contacts made by PS residues could explain the relatively small ΔΔ*G_PS(Np-Rp)_* values, yet it does not explain the larger effects on ΔΔ*G^‡^*.

Φ-values outside the classical range of 0 to 1 are the result of opposite or larger energetic effects in the TS than in the native state, although the microscopic basis for this is not certain. One hypothesis is that these unusual Φ-values arise from alternative flow channels down the folding funnel [38], with alternative folding paths (and thus a different TS to be crossed) becoming more predominant upon introduction of a point mutation. While the notion of alternative flow channels is consistent with the view of a funneled folding landscape, the only supporting evidence to date has come from native-centric lattice Gō models [38]. An alternative interpretation is that unusual Φ-values can arise when side-chains form non-native contacts in the TS [39], [40]. Both experiments and simulations have shown that non-native interactions can accelerate or decelerate folding [39]–[43]. Suspected non-native interactions were found to involve both hydrophobic and electrostatic interactions, and these can stabilize or destabilize the TS [26], [40], [44]. In addition to non-native interactions, unusual Φ-values may arise upon mutation of a group that experiences different conformational strain in the TS and native state. Mutations that change the size of hydrophobic side-chains can stabilize the TS (by optimizing side-group packing) while destabilizing the native state, due to the different compactness of the TS, thereby producing negative Φ-values [30]. Similarly, mutations that stabilize the native state yet destabilize the TS due to differences in conformational strain also produce negative Φ-values [45]. Conformational strain present in the TS but not in the native state can also give rise to Φ-values >1 [46]. In PS-pepsin the V4A mutation was stabilizing to the native state and destabilizing to the TS, suggestive of a slightly frustrated or overly-packed native state and a more optimally packed TS. In fact, all the anomalous Φ-values for PS-pepsin (both negative and positive) resulted from ΔΔ*G^‡^* > ΔΔ*G_PS(Np-Rp)_*.

The latter result is consistent with the finding that the PS has picomolar affinity for the TS compared to nano- and micromolar affinity for Np and Rp, respectively [7]. It seems likely that the PS has achieved this higher affinity for the TS via a concerted optimization of side-chain packing, hydrogen bonding and electrostatics, as evidenced by polar, hydrophobic and charged groups selectively stabilizing the TS (**Fig 3A**). Furthermore, this concerted optimization likely involves both non-native and strengthened native contacts, given the range of interactions involved. In the native fold [47], PS residues R8, R13 and K36 form ion pairs with pepsin residues E13, D11 and D32, D215, respectively, and these may be optimized in PS-TS. S11 is flanked on either side by R8 and R13 and thus may influence the strength of these interactions. V4 and L6 are likely optimally packed in the TS compared to in PS-Rp and PS-Np, in line with previous evidence that non-native packing accelerates folding [39]–[43]. Further insight into the nature of these contacts may be gleaned from future studies involving double-mutant cycles (*e.g*., to study the influence of ion-pairs) and the systematic reduction of side-chain size (*e.g*., Val → Ala → Gly), and both approaches have been used previously [20], [26], [27], [40].

Coarse-grain simulations have been used to understand the nature of non-native interactions in small, single-domain proteins lacking stable folding intermediates [40], [48], [49]. To our knowledge, such approaches have not yet been applied to larger proteins, although the folding of DehI, a 311-residue protein with a knotted-fold, was simulated using a native-centric Gō model that did not include non-native contacts [50]. It would be challenging, but not impossible, to use similar coarse-grain approaches to model the folding of pepsinogen (370 residues). Using simulation to gain insight into the nature of the non-native contacts formed within PS-TS would be greatly facilitated by knowing the high-resolution structure of PS-Rp. This would require characterizing the PS-Rp complex before it folded to PS-Np. The data presented here (**Fig 3A**) indicate that PS-Rp could be ‘trapped’ for further structural analysis by using a PS with a double mutation (such as PS_I17A/F25A_), which would be expected to shift the folding equilibrium from PS_I17A/F25A_-Np to PS_I17A/F25A_-Rp. This hypothesis was confirmed recently using ^1^H-^15^N TROSY NMR to show that PS_I17A/F25A_-Rp is structurally very similar to Rp alone [51].

Only I17A (Φ = 0) and F25A (Φ = 1.1) gave typical Φ-values, indicating that I17 adopts native-like structure after formation of the TS, while F25 adopts native-like structure during formation of the TS. F25 is located on the second α-helix of the PS, which runs across the top of the active site cleft in pepsinogen (**Fig's 2A and S2**), the C-terminus of which (residue 29) marks the end of the conserved A1 propeptide motif [34]. Given that F25A yielded a Φ-value close to 1 indicates that this α-helical segment may be structured in the TS, suggesting its importance to PS-catalyzed folding.

### PS stabilizes a late-stage transition between compact misfolded and native states

For pepsin [7], [15], αLP4 [8], and SGPB [5], the PS catalyzes folding from a compact, well-structured denatured state, indicating that the PS acts at a late stage in the folding process. In the case of pepsin, Rp was characterized by a Δ*G*_unf_ of 5.8 kcal/mol, a 10% increase in unordered secondary structure and identical tertiary structure to Np (both yield *R*_g_ ∼20 Å) [15]. Similarly, the stable denatured state of αLP was found to have secondary and tertiary structures intermediate between the native and unfolded forms, with a 9% increase in unordered secondary structure and a 40% increased hydrodynamic radius [4]. The intermediate αLP gave a Δ*G*_unf_ of 1 kcal/mol, which was 4 kcal/mol more stable than the native state [4]. The SGPB intermediate was characterized by a Δ*G*_unf_ of 0.5 kcal/mol but was less stable than the native state by 0.8 kcal/mol [5].

Given the unique properties of Rp, a thermodynamically stable [7], rigid [16], native-like yet inactive form, it is reasonable to suppose that Rp is a late-stage misfolded state that lacks the correct domain-domain interactions found in Np. The active site cleft of Np is formed between the N- and C-terminal lobes (**Fig 2A**), and it is possible that the PS catalyzes the correct formation of inter-domain contacts. This scenario is consistent with the relatively small changes in secondary and tertiary structure that accompany the Rp to Np transition [15]. This picture is also consistent with the concept of independent foldon units (in this case the N- and C-terminal domains), which fold independently, followed by a rate-limiting docking step [52]. In the case of pepsin, and perhaps other zymogen-derived proteins, the rate-limiting docking of domains is slow enough that a PS is required to act as a foldase.

With the discovery of PS-assisted folding in a few evolutionarily unrelated serine peptidases, it was suggested that PS-catalyzed folding has developed through convergent evolution [11], [13], a notion supported by the similarities that exist in the folding mechanisms of pepsin and αLP. Interestingly, the PS of pepsinogen is 44 residues in length whereas the PS domains for αLP and SGPB are much longer, at 166 and 76 residues, respectively. The PS-catalyzed folding of pepsin yields a folding rate enhancement (*k*_cat_/*k*_non-cat_) far greater than that of SGPB, yet is less than that of αLP (**Fig 1AB**). Thus, PS length is not necessarily correlated with the power of the PS as a folding catalyst. This comparison also highlights that the pepsin PS is a highly efficient folding catalyst: at a mere 44 residues in length it provides substantial TS stabilization per residue (**Fig 3A**), likely via a high density of strengthened native and/or non-native contacts.

Mutations to the PS of αLP were also found to have a greater impact on the PS-catalyzed folding rate than on binding the native or intermediate states, determined by measuring *K*_i_ and *K*_M_ values, respectively [53]. PS mutations Y26F, E30A, and Y26F/E30A resulted in essentially no change in *K*_i_ or *K*_M_ while *k*_cat_ was reduced by a factor of 10, 2, and 60, respectively [53]. These findings reflect the fact that the PS binds most tightly to the folding TS for αLP, and such interactions may involve non-native contacts, similarly as for pepsin.

### The PS may ‘buffer’ the folding landscape via non-native interactions

The physiological basis for the existence of PS-catalyzed folding as a folding mechanism is not clear. For αLP it was shown that the PS aids in the formation of a kinetically trapped native state that is highly rigid, thus conferring enhanced resistance to proteolysis for this extracellular serine peptidase [5], [54]. In contrast, pepsin was shown to have a relatively flexible native conformation [16], and it is reasonable to conclude that pepsin has evolved a different mechanism of resistance to proteolysis: Np is kinetically stable at acidic pH where most exogenous proteases would be inactivated, allowing for digestion by pepsin. Pepsin and αLP are both kinetically trapped and are thermodynamically metastable or unstable, respectively, yet these features may not be related, given that many thermodynamically stable proteins are at least as, if not more, kinetically stable than pepsin or αLP [5], [55], [56]. PS-catalyzed folding is not required to generate a kinetically stable fold.

Given the above considerations, there must be a more universal role for PS-catalyzed folding. We hypothesize that PS-catalyzed folding allows for more destabilizing contacts to accumulate in the native fold, thereby allowing for a greater search of evolutionary space that would otherwise be restricted by the loss of stability (*e.g*., this could result in novel functions/substrate specificity). This is akin to the ‘buffering capacity’ that the chaperonins GroEL/ES were shown to provide in enhancing protein evolvability [57], [58]. Indeed, αLP [8] and pepsin [7] are thermodynamically unstable/metastable native states that would not exist without PS-catalyzed folding. In such a scenario, the PS acts to buff or smooth [59] the folding landscape via non-native interactions, catalyzing the folding to a thermodynamically stable PS-native state complex. Upon removal of the PS, these stabilizing contacts are no longer available in the unfolding TS; thus, the unfolding barrier is increased yielding a kinetically trapped native state.

## Materials and Methods

### Materials

Synthetic peptides were obtained from CanPeptide Inc. (Pointe-Claire, QC, Canada), and were more than 95% pure as judged by LC-MS. Peptides corresponding to the 44-residue PS domain of pepsinogen were obtained in wild-type and the following single mutant forms, in which the wild-type residue was replaced with alanine: V4A, L6A, S11A, R13A, I17A, F25A, and K36A. Porcine pepsin A (EC 3.4.23.1) was purchased from Sigma (St. Louis, MO, USA) and used without further purification. Protein solutions were prepared by mass (wt/vol) and the concentrations were determined from the absorbance at 280 nm, using extinction coefficients of 1490 M^−1^cm^−1^ for the PS peptides and 52,830 M^−1^cm^−1^ for pepsin, estimated using the ProtParam tool [60]. Rp samples were prepared by first denaturing pepsin by making a 20 mg/ml solution in 30 mM NaOH, with a final pH of 8, yielding alkaline denatured pepsin. Rp was then obtained by diluting an aliquot of the alkaline denatured protein to 0.35 mg/ml in 20 mM acetic acid/NaOH buffer at pH 5.3.

### Sequence alignment

Sequences similar to that of porcine pepsinogen were identified using an NCBI-BLASTp search [61], and the results were limited to the 100 top scoring, non-redundant sequences. Multiple sequence alignment of this group was then performed using CINEMA 5 [62], in which a breakpoint was added to isolate the alignment of the PS domain from that of the mature domain.

### PS-catalyzed folding of Rp to Np

PS-catalyzed folding was carried out by combining 1 µM Rp and 30 µM PS, in a volume of 100 µl, at pH 5.3 (20 mM acetic acid/NaOH with 100 mM NaCl) and 15°C. Aliquots were taken at several time intervals, diluted 20-fold in 50 mM phosphoric acid buffer, pH 1.2, incubated for 5 min at 25°C and assayed for Np activity using the KPAEFF(NO_2_)AL substrate. The recovery of Np activity with time, *t*, was fit with a monoexponential function (*y* = *a* – *b*exp(–*k*_f_ *t*)) to obtain the PS-catalyzed folding rate constant, *k*_f_. Under these conditions, PS binding to Rp reached equilibrium within the dead-time of mixing (<8 sec), as judged by Trp-fluorescence.

### PS binding to Rp

The change in intrinsic tryptophan fluorescence of pepsin was used to measure the binding of PS to Rp and to determine the dissociation constant, *K*_d_. Rp solutions were diluted to between 0.6 and 1.2 µM in 20 mM acetic acid/NaOH buffer, pH 5.3, and mixed with various amounts of PS. After incubating the samples at 20°C for ≥10 minutes, the intrinsic tryptophan fluorescence was measured using a PTI spectrofluorophotometer (Photon Technology International, Inc., Birmingham NJ, USA), with excitation at 295 nm and emission measured at 315 nm. The change in fluorescence, Δ*F*_i_, at each PS concentration, [*PS*], was normalized relative to the maximum change, Δ*F*_max_, and fit according to
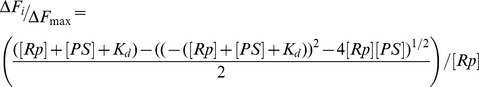
(1)where [*Rp*] is the total concentration of pepsin.

PS binding to Rp was measured in buffer without added 100 mM NaCl in order to obtain more accurate measurements of *K*_d_, owing to a larger change in signal in the absence of salt. Additionally, without added 100 mM NaCl it was possible to isolate the PS-Rp binding step from the catalyzed folding step thereby improving the determination of *K*_d_. Without 100 mM NaCl, no generation of protease activity was observed on a timescale of 0–2 hours (data not shown, and [7]), indicating that the PS binds Rp yet does not catalyze folding to Np. However, ^1^H-^15^N TROSY NMR experiments [51] indicated that PS-Rp does fold to the native complex in the absence of 100 mM NaCl, but over longer timescales of days (**Fig S7**).

### PS binding to Np

PS binding to Np was determined by using the PS as a competitive inhibitor and measuring the inhibition constant, *K*_i_. Hydrolysis of the KPAEFF(NO_2_)AL substrate was measured by following the decrease in absorbance at 300 nm using a Biochrom Ultrospec 3100pro UV-Vis spectrophotometer (Biochrom Ltd., Cambridge, England) in 20 mM acetic acid/NaOH buffer, pH 5.3, containing 100 mM NaCl. Np samples were diluted to 10 nM and incubated with PS for 5 min at 20°C and assayed for activity. The reaction rates were normalized to the activity in the absence of PS and the data fit using the competitive inhibitor form of the Michaelis-Menten equation
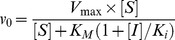
(2)where *v_0_* is the initial reaction rate, *V*_max_ is the maximum reaction rate, [*S*] is the substrate concentration (fixed at 0.1 mM), [*I*] is the inhibitor concentration, and *K*_M_ is the Michaelis constant.

### CD spectroscopy

CD data were collected using a Jasco J-810 spectropolarimeter (Jasco corp., Tokyo, Japan), over a wavelength range of 250 nm to 190 nm with a 1 nm resolution, 100 nm/min scan rate, 0.25 s response time and four-fold accumulation of scans. PS_wt_ was diluted to 0.1 mg/ml in either pure water or 20 mM acetic acid/NaOH buffer at pH 5.3, with and without 100 mM NaCl, and loaded into a cell with a 0.1 cm path length. Background spectra were subtracted and the sample spectra converted to units of mean residue ellipticity, *MRE*, using *MRE*  =  *MRW* ×*θ*_λ_/(10×*d*×*c*), where *MRW* is the mean residue weight (molecular weight/number of residues), *θ*_λ_ is the measured ellipticity at a particular wavelength (degrees), *d* is the pathlength (0.1 cm) and *c* is the protein concentration (g/cm^3^).

### Calculation of Φ-values from PS-catalyzed folding and binding constants

The change in stability of the folding transition state upon mutation was calculated using
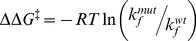
(3)where *k*_f, wt_ and *k*_f, mut_ are the PS-catalyzed folding rate constants of the wild-type and mutant PS peptides. The change in equilibrium stability of PS-Np relative to PS-Rp upon mutation of the PS (ΔΔ*G_PS(Np-Rp)_*) was determined as the difference between the changes in binding energies, using
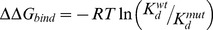
(4)and
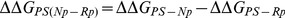
(5)


Here, ΔΔ*G_bind_* refers to either ΔΔ*G_PS-Rp_*, determined from *K*_d_ values, or ΔΔ*G_PS-Np_*, determined from *K*_i_ values. The Φ-value for each mutant corresponds to eq 3 divided by eq 5. Additional details are included in the supporting information section (**Text S1**).

## Supporting Information

Figure S1 **PS-catalyzed folding approach to Φ-value analysis.** The effect of a mutation on each step of the folding landscape was determined separately by measuring PS-catalyzed folding and binding affinities rather than directly measuring the equilibrium stability of PS-Np relative to PS-Rp, ΔΔ*G_PS(Np-Rp)_*. The relative changes in binding affinities gave ΔΔ*G_PS(Np-Rp)_*, while ΔΔ*G^‡^* was obtained directly from the relative folding rates.
(TIF)

Figure S2 **Structure of the PS domains of pepsinogen (PDB: 3PSG) and progastricsin (PDB: 1HTR).** Ribbon diagram showing select residues of pepsinogen (red side chains, black backbone) and progastricsin (blue side chains, grey backbone), starting from the N-terminus, pepsinogen numbering: V4, L6, R8, S11, R13, I17, F25 and K36. The overall fold is very similar with an average RMSD of 1.24 Å, while particularly for the conserved residues L6 and R13 and the semi-conserved V4, S11 and K36, the structures are identical, with RMSD <1 Å.
(TIF)

Figure S3 **Sequence alignment of the PS domain of pepsinogen with the nearest 100 sequences.** The first sequence from the top is porcine pepsinogen (PEPA_PIG).
(TIF)

Figure S4 **Determination of** ***k*** **_f_,** ***K*** **_d_ and** ***K*** **_i_ for PS-catalyzed folding, PS-Rp binding and PS-Np binding, respectively.** Data and fit curves are the same as those shown in Fig 2BCD in the main text, and are plotted for each individual wt- and mutant PS for clarity.
(TIF)

Figure S5 **Temperature dependence of PS_wt_-catalyzed folding of Rp to Np.** Rates were measured at 0, 5, 10 and 15°C, using recovery of Np activity. Data points are the mean ± SD of at least three determinations. The linear fit is also indicated.
(TIF)

Figure S6 **CD spectra of prosegment.** Far-UV CD spectra of PS_wt_, in ddH_2_O and in 20 mM acetic acid/NaOH, at pH 5.3, with and without 100 mM NaCl added. A negative band at 198 nm is characteristic of random coil structure [Sreerama N, Venyaminov SY, Woody RW (2000) Estimation of protein secondary structure from circular dichroism spectra: inclusion of denatured proteins with native proteins in the analysis. Anal Biochem 287: 243–251].
(TIF)

Figure S7 **PS-Rp folds to an identical native conformation with and without added salt.** ^1^H-^15^N TROSY NMR spectra were collected for samples of PS combined with Rp in buffers (A) containing 100 mM NaCl and (B) without added 100 mM NaCl. (C) Overlay of the two spectra. The buffer was 20 mM NaOAc pH 5.3 with 10% D_2_O, at 22°C. The NMR experimental details were published previously [51].
(TIF)

Text S1 **Calculation of Φ-values from PS-catalyzed folding and binding constants.**
(DOCX)

Text S2 **Temperature dependence of PS-catalyzed folding.**
(DOCX)
