# Supplementary figures and images for: Conserved Prosegment Residues Stabilize a Late-Stage Folding Transition State of Pepsin Independently of Ground States
Source: PLoS One. 2014 Jul 1;9(7):e101339. doi: 10.1371/journal.pone.0101339 (PMC4077824; doi:10.1371/journal.pone.0101339)

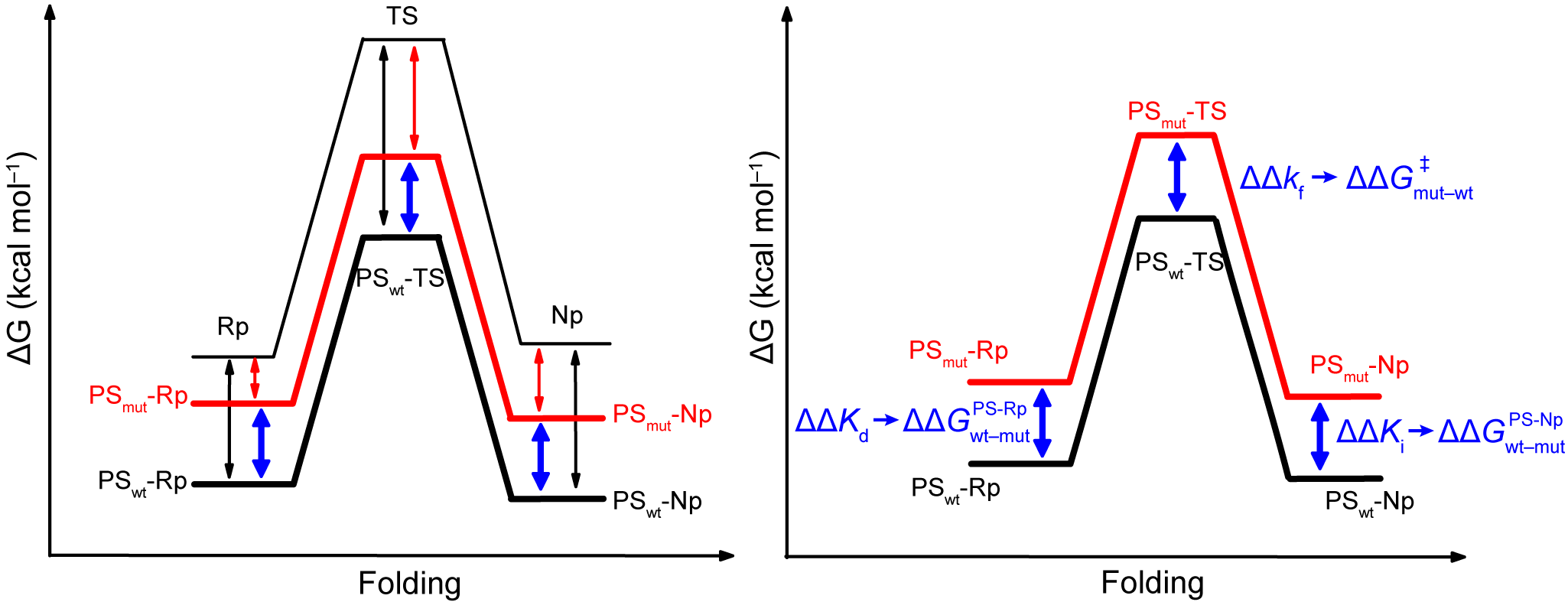

Supplement: Figure S1 — PS-catalyzed folding approach to Φ-value analysis. The effect of a mutation on each step of the folding landscape was determined separately by measuring PS-catalyzed folding and binding affinities rather than directly measuring the equilibrium stability of PS-Np relative to PS-Rp, ΔΔGPS(Np-Rp). The relative changes in binding affinities gave ΔΔGPS(Np-Rp), while ΔΔG‡ was obtained directly from the relative folding rates. (TIF) [file pone.0101339.s001.tif]

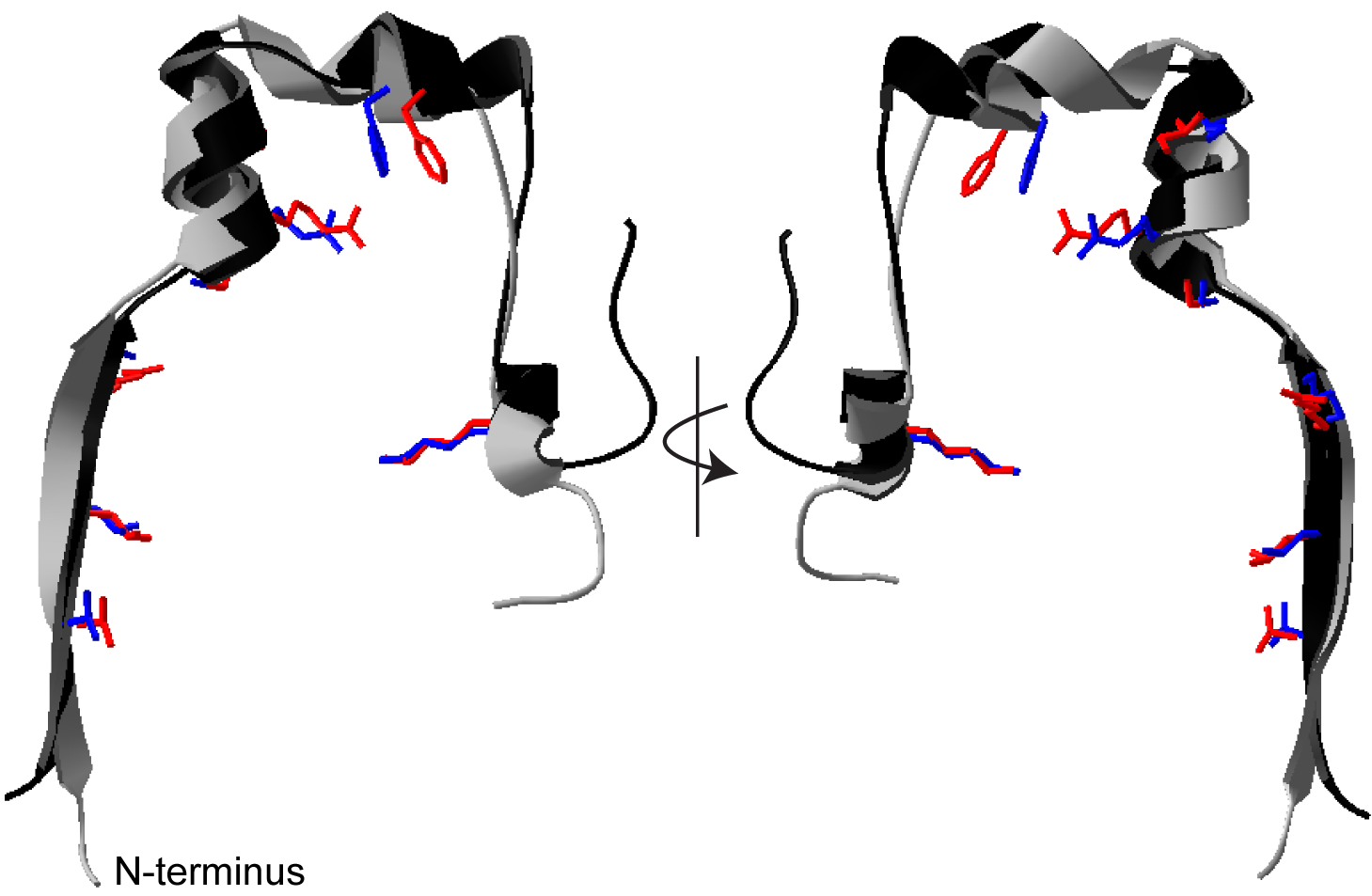

Supplement: Figure S2 — Structure of the PS domains of pepsinogen (PDB: 3PSG) and progastricsin (PDB: 1HTR). Ribbon diagram showing select residues of pepsinogen (red side chains, black backbone) and progastricsin (blue side chains, grey backbone), starting from the N-terminus, pepsinogen numbering: V4, L6, R8, S11, R13, I17, F25 and K36. The overall fold is very similar with an average RMSD of 1.24 Å, while particularly for the conserved residues L6 and R13 and the semi-conserved V4, S11 and K36, the structures are identical, with RMSD <1 Å. (TIF) [file pone.0101339.s002.tif]

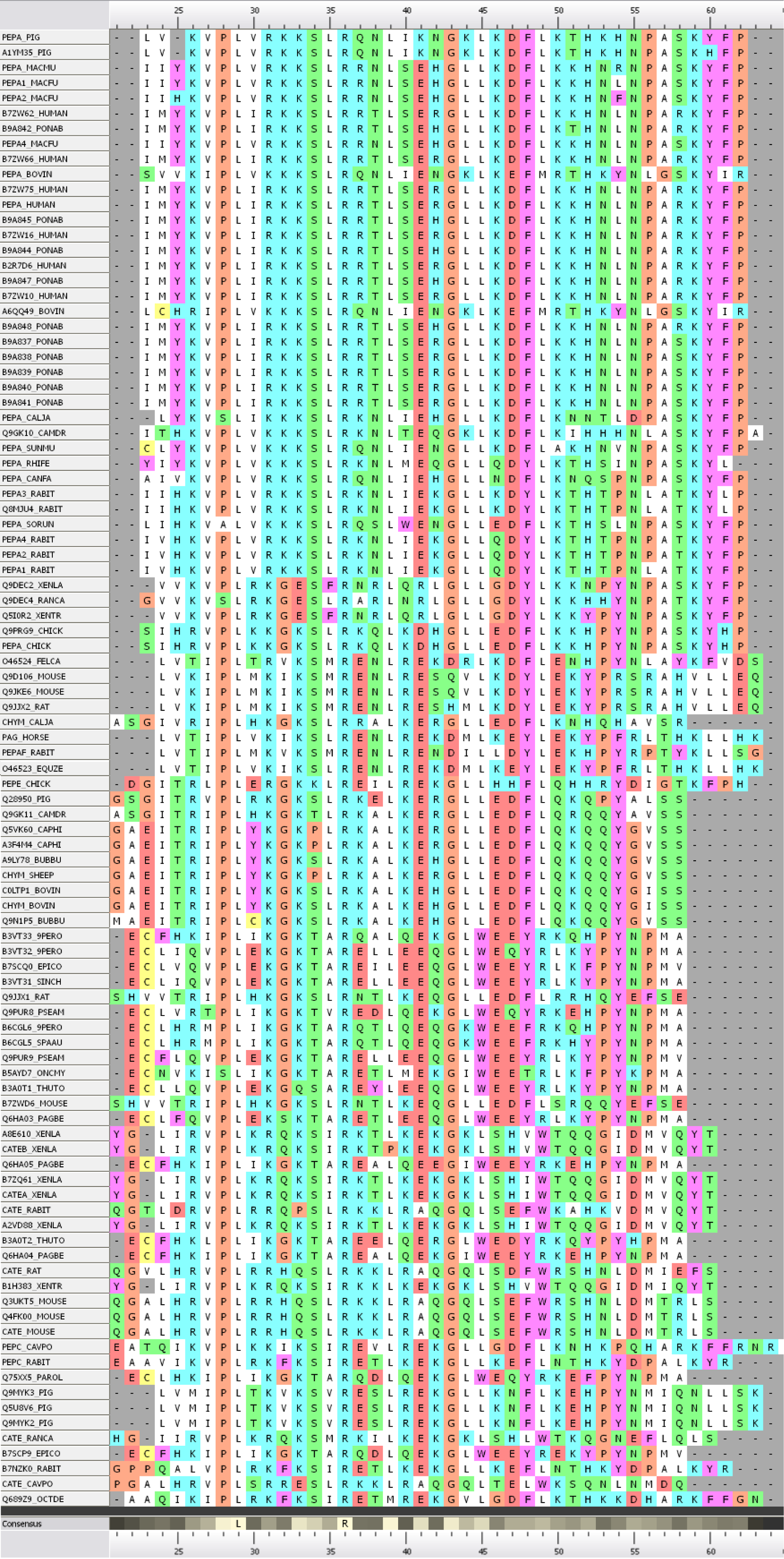

Supplement: Figure S3 — Sequence alignment of the PS domain of pepsinogen with the nearest 100 sequences. The first sequence from the top is porcine pepsinogen (PEPA_PIG). (TIF) [file pone.0101339.s003.tif]

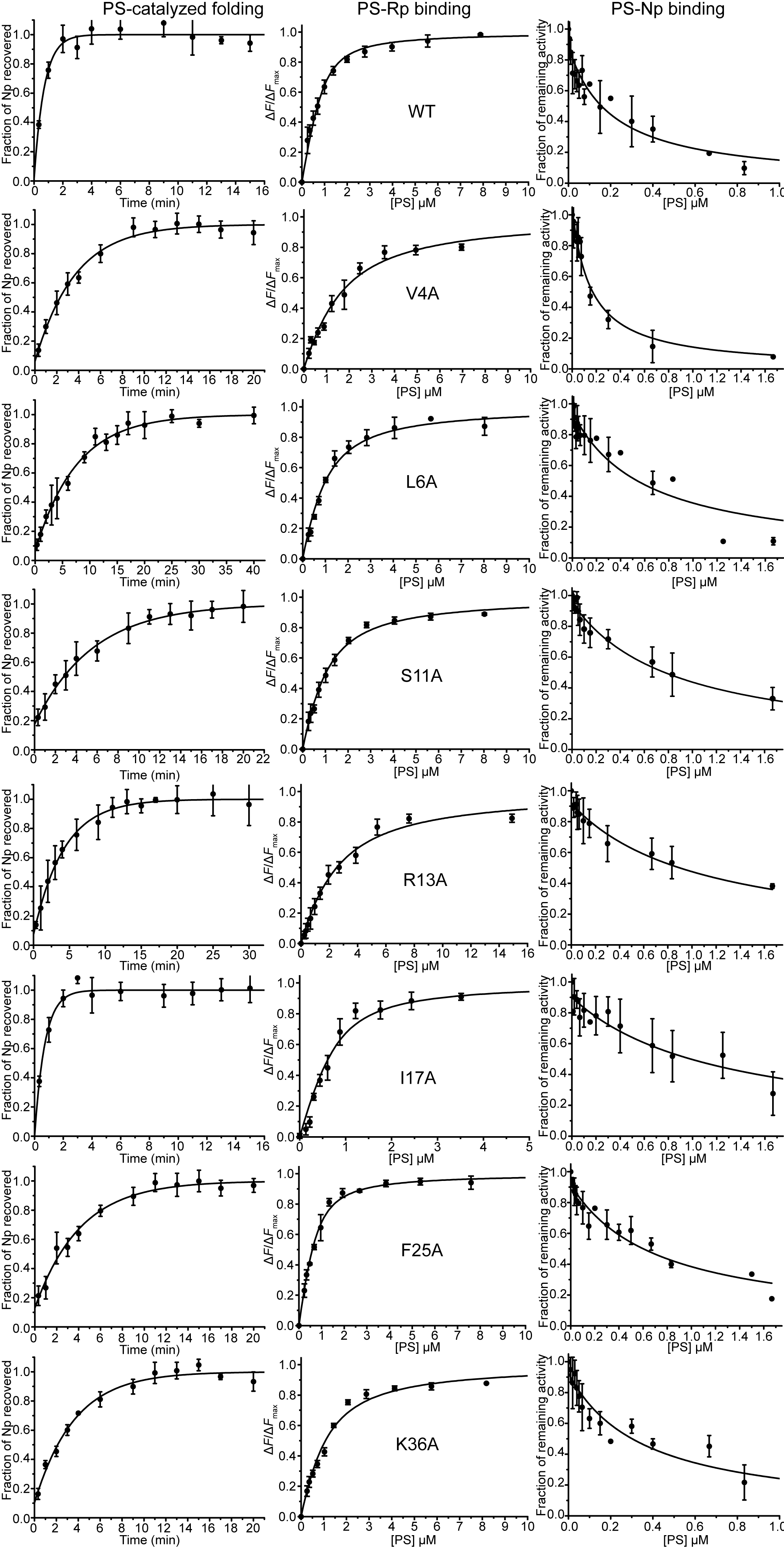

Supplement: Figure S4 — Determination of k f, K d and K i for PS-catalyzed folding, PS-Rp binding and PS-Np binding, respectively. Data and fit curves are the same as those shown in Fig 2BCD in the main text, and are plotted for each individual wt- and mutant PS for clarity. (TIF) [file pone.0101339.s004.tif]

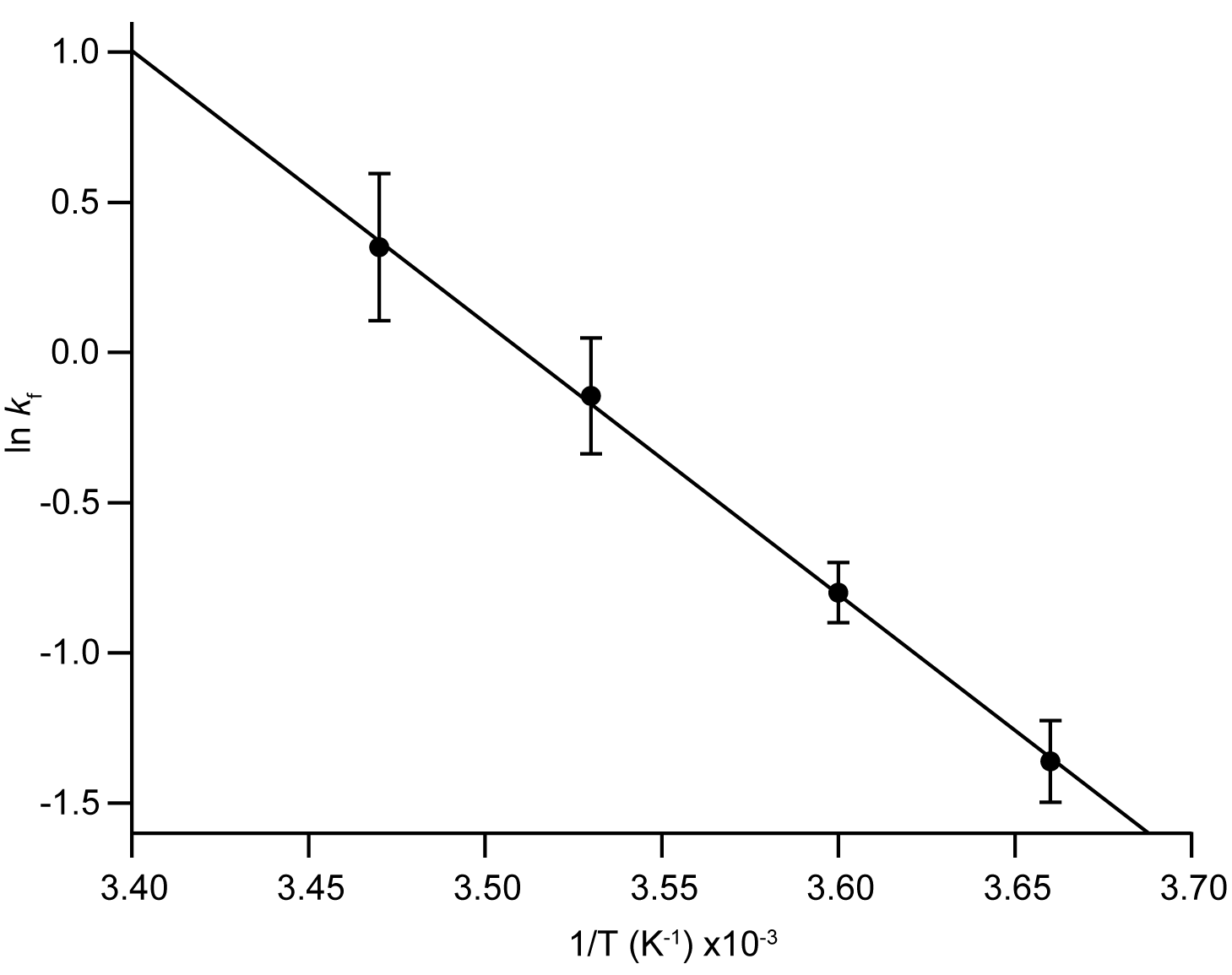

Supplement: Figure S5 — Temperature dependence of PSwt-catalyzed folding of Rp to Np. Rates were measured at 0, 5, 10 and 15°C, using recovery of Np activity. Data points are the mean ± SD of at least three determinations. The linear fit is also indicated. (TIF) [file pone.0101339.s005.tif]

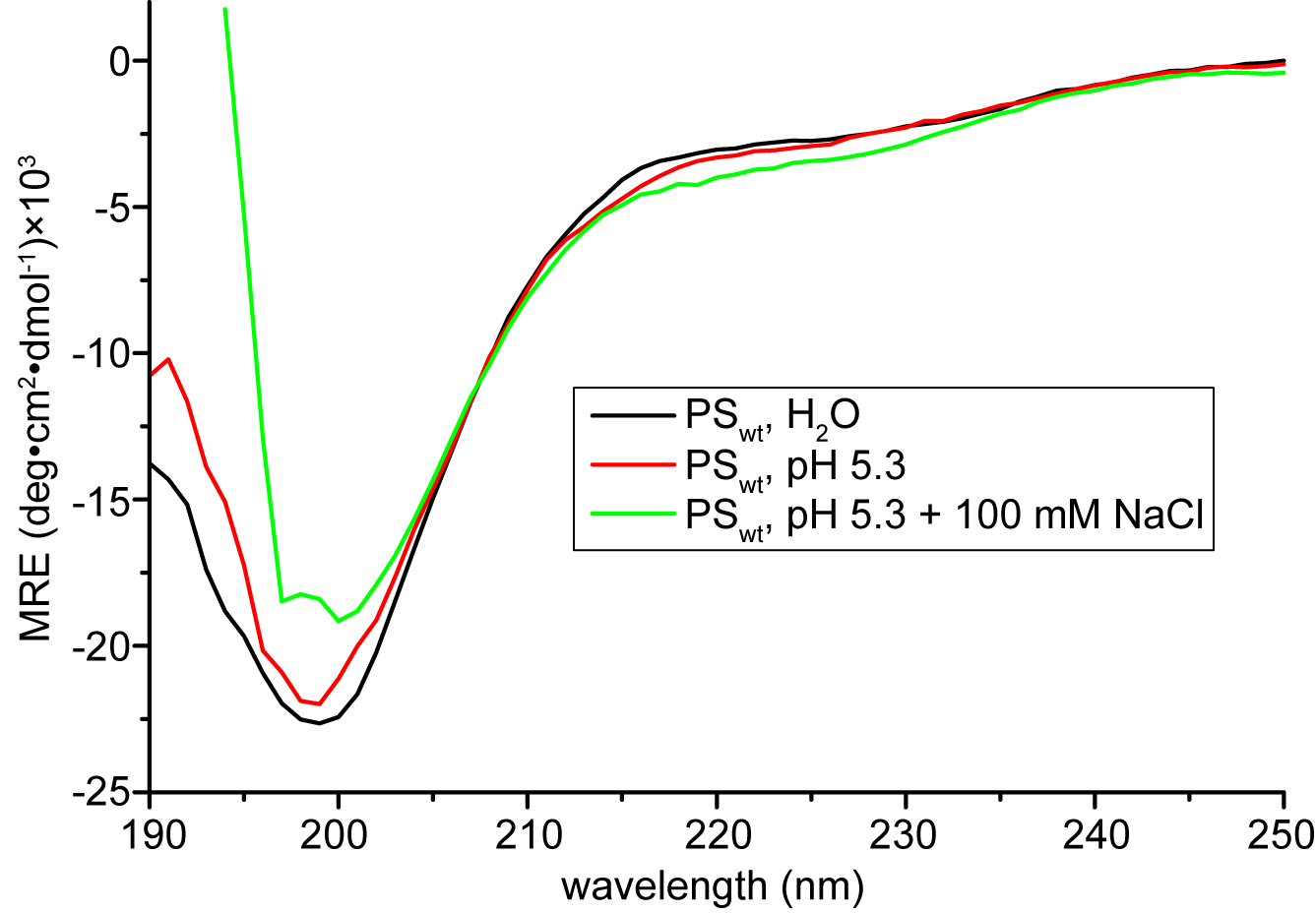

Supplement: Figure S6 — CD spectra of prosegment. Far-UV CD spectra of PSwt, in ddH2O and in 20 mM acetic acid/NaOH, at pH 5.3, with and without 100 mM NaCl added. A negative band at 198 nm is characteristic of random coil structure [Sreerama N, Venyaminov SY, Woody RW (2000) Estimation of protein secondary structure from circular dichroism spectra: inclusion of denatured proteins with native proteins in the analysis. Anal Biochem 287: 243–251]. (TIF) [file pone.0101339.s006.tif]

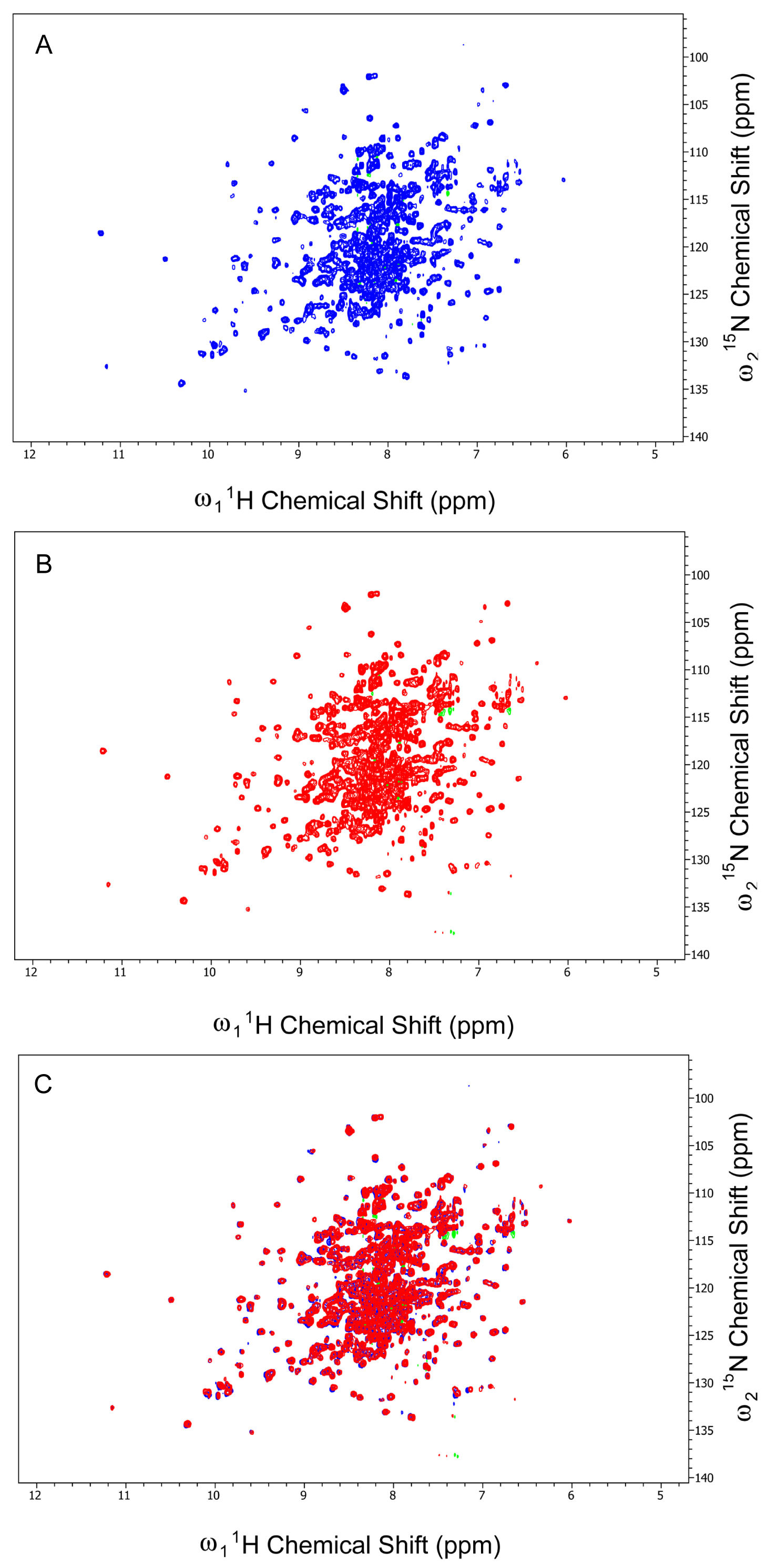

Supplement: Figure S7 — PS-Rp folds to an identical native conformation with and without added salt. 1H-15N TROSY NMR spectra were collected for samples of PS combined with Rp in buffers (A) containing 100 mM NaCl and (B) without added 100 mM NaCl. (C) Overlay of the two spectra. The buffer was 20 mM NaOAc pH 5.3 with 10% D2O, at 22°C. The NMR experimental details were published previously [51]. (TIF) [file pone.0101339.s007.tif]
