# Supplementary material for: Conserved Prosegment Residues Stabilize a Late-Stage Folding Transition State of Pepsin Independently of Ground States
Source: PLoS One. 2014 Jul 1;9(7):e101339. doi: 10.1371/journal.pone.0101339 (PMC4077824; doi:10.1371/journal.pone.0101339)
Supplement: Text S1 — Calculation of Φ-values from PS-catalyzed folding and binding constants. (DOCX) [file pone.0101339.s008.docx]

**Text S1**

**Calculation of Φ-values from PS-catalyzed folding and binding constants**

As outlined below, ∆*G*_bind_ values are used to calculate *∆∆G*_PS(Np-Rp)_, which is the change in the equilibrium free energy difference between PS-Np and PS-Rp upon mutation of the PS (for a diagram of the folding reaction, see **Fig S1**)

$\Delta\Delta G_{bind}=\Delta G_{bind}^{wt}-\Delta G_{bind}^{mut}$ (S1)

ΔΔ*G_bind_* can refer to either ∆∆*G*^PS-Np^_(wt-mut)_ or *∆∆G*^PS-Rp^_(wt-mut)_. Also, we can write ∆*G*_bind_ in terms of its components

$\Delta G_{bind}=\Delta G_{PS}+\Delta G_{pep\sin}+\Delta G_{solvent}$ (S2)

where ∆*G*_PS_, ∆*G*_pepsin_ and ∆*G*_solvent_ refer to the change in free energy of the PS, pepsin and the solvent upon PS binding to pepsin, respectively. Combining equations S1 and S2 gives

$\Delta\Delta G_{bind}=(\Delta G_{PS}+\Delta G_{pep\sin}+\Delta G_{solvent})_{wt}-(\Delta G_{PS}+\Delta G_{pep\sin}+\Delta G_{solvent})_{mut}$ (S3)

The contribution of each component to ∆∆*G*_bind_ will depend on the particular mutation (e.g., nature of substitution, location within folded structure, solvent accessibility). This is the general case for protein engineering analysis, in which the effects of mutations on changes in equilibrium and activation energies are the result of many individual interaction energy terms, including solvent terms, as previously explained in detail [Fersht AR, Matouschek A, Serrano L (1992) The folding of an enzyme I. Theory of protein engineering analysis of stability and pathway of protein folding. J Mol Biol 224: 771‒782]. Most importantly, there is no requirement to separate ΔΔ*G_bind_* into its components in order to proceed with analysis as we are interested in the overall change in equilibrium stability of the PS-pepsin complex (*∆∆G*_PS(Np-Rp)_)

$\Delta\Delta G_{PS(Np-Rp)}=\Delta\Delta G_{(wt-mut)}^{PS-Np}-\Delta\Delta G_{(wt-mut)}^{PS-Rp}$ (S4)

This bimolecular approach to Φ-value analysis of protein folding is qualitatively identical to Φ-value analysis as it was originally applied to the study of enzyme catalysis. As outlined previously [Fersht AR], changes in the free energy of enzyme (E) binding to substrate (S) or product (P) are used to determine the change in free energy difference between E-P and E-S upon mutation, *∆∆G*^EP-ES^_(wt-mut)_, where

$\Delta\Delta G_{(wt-mut)}^{EP-ES}=\Delta\Delta G_{(wt-mut)}^{E-P}-\Delta\Delta G_{(wt-mut)}^{E-S}$ (S5)

As these are at the scale of global energy terms, equations S4 and S5 are equivalent and PS-catalyzed folding can be treated similarly as enzyme catalysis, in which the PS is the foldase and the denatured and natively folded protein are the substrate and product, respectively. However, protein folding reactions are more complicated to interpret at the level of individual interaction energy terms, which are more numerous [Fersht AR].

Changes in the binding energies between PS-Rp and PS-Np upon PS mutation can then be used to determine Φ-values with the following considerations:

1. Free PS is unstructured (see **Fig S6**), so Ala mutations are expected to have a negligible effect on the stability and conformation of free PS.

2. Changes in binding and folding upon mutation can be localized to the loss of interactions in the immediate vicinity of the truncation mutation (i.e., larger residue mutated to Ala), and not due to gross conformational changes of the PS or changes in the binding/folding mechanism (consider as strong evidence that all of the mutant PS’s readily catalyze folding to the native, active form).

3. Changes in PS-Rp and PS-Np binding energies upon mutation (∆∆*G*^PS-Np^_(wt-mut)_ and *∆∆G*^PS-Rp^_(wt-mut)_) are measured directly, while the change in the PS-TS binding energy (∆∆*G*^PS-TS^_(wt-mut)_) is the sum of ∆∆*G*^PS-Rp^_(wt-mut)_ and ∆∆*G*^‡^_(wt-mut)_.

4. Φ-values are calculated using

$\Phi=\frac{\Delta\Delta G_{mut-wt}^{PS-TS}-\Delta\Delta G_{wt-mut}^{PS-Rp}}{\Delta\Delta G_{wt-mut}^{PS-Np}-\Delta\Delta G_{wt-mut}^{PS-Rp}}=\frac{\Delta\Delta G_{mut-wt}^{\ddagger}}{\Delta\Delta G_{wt-mut}^{PS(Np-Rp)}}$ (S6)

As in all cases of Φ-value analysis, the energetic perturbations of mutation are measured relative to one state (generally the unfolded state, but in theory it can be any state). In this case, all measurements are relative to PS-Rp.
