# Supplementary material for: Conserved Prosegment Residues Stabilize a Late-Stage Folding Transition State of Pepsin Independently of Ground States
Source: PLoS One. 2014 Jul 1;9(7):e101339. doi: 10.1371/journal.pone.0101339 (PMC4077824; doi:10.1371/journal.pone.0101339)
Supplement: Text S2 — Temperature dependence of PS-catalyzed folding. (DOCX) [file pone.0101339.s009.docx]

**Text S2**

**Temperature dependence of PS-catalyzed folding**

PS-catalyzed folding could be measured only up to 15 ºC for PS_wt_, as the reaction was too fast at higher temperatures. However, it was found that the natural logarithm of the PS_wt_-catalyzed folding rate constant varied linearly with 1/T from 0 ºC to 15 ºC, as shown in **Fig S5**. Thus, PS-catalyzed folding of pepsin followed Arrhenius temperature-dependence within the relevant temperature range, indicating that the folding activation barrier remains constant. Given the linear temperature dependence it could be reasonably assumed that changes in activation energies (mutant – wild-type) would be unchanged from 15 ºC to 20 ºC, and so could be compared directly with changes in binding energies (wild-type – mutant) that were determined at 20 ºC.
